# Successful Repair of a Left Pulmonary Artery Sling in an Adult With Airway Obstruction

**DOI:** 10.1016/j.atssr.2025.09.010

**Published:** 2025-10-08

**Authors:** Matthew D. Warren, Eric V. Krieger, David Mauchley, Michael S. Mulligan, Samuel G. Rayner

**Affiliations:** 1 Division of Cardiology, Department of Medicine, University of Washington, Seattle, Washington; 2 Division of Cardiothoracic Surgery, Department of Surgery, University of Washington, Seattle, Washington; 3 Division of Pulmonary, Critical Care and Sleep Medicine, Department of Medicine, University of Washington, Seattle, Washington

## Abstract

Left pulmonary artery (LPA) sling is a rare congenital vascular anomaly that typically manifests in infancy with symptomatic tracheal and esophageal compression. Unrepaired patients are rarely encountered in adulthood. This case describes a 63-year-old woman with lifelong symptoms related to an unrepaired LPA sling and progressive airway obstruction. Testing confirmed severe tracheal and esophageal compression, and she underwent LPA translocation. Following surgery, her symptoms improved, with resolution of tracheal compression on imaging and normalization of pulmonary function testing.

Left pulmonary artery (LPA) sling is a rare congenital vascular abnormality, accounting for approximately 5% of diagnosed vascular ring syndromes.^1^ In this condition, the LPA arises aberrantly from the right pulmonary artery such that the LPA traverses between the trachea and esophagus and compresses these structures (Figure 1). If not diagnosed prenatally, it typically manifests in infancy with respiratory distress or dysphagia, most often requiring surgical repair. Less commonly, symptoms are mild or even absent, delaying discovery.^2^^,^^3^ Few reports of symptomatic presentation in adults exist, and only a single report of adult operative repair of an LPA sling for the purpose of symptoms (dysphagia) exists, with a second report detailing a repair undertaken at the time of tumor resection.^4^^,^^5^ The efficacy of adult surgical repair for airway compression due to LPA sling is unknown, and pulmonary function testing before and after intervention has not been reported. Here, we describe a 63-year-old woman with lifelong respiratory symptoms related to an unrepaired LPA sling who experienced symptomatic progression later in life. She underwent operative repair of the LPA sling with improvement in her symptoms, examination findings, imaging, and pulmonary function testing.

**Figure 1:**
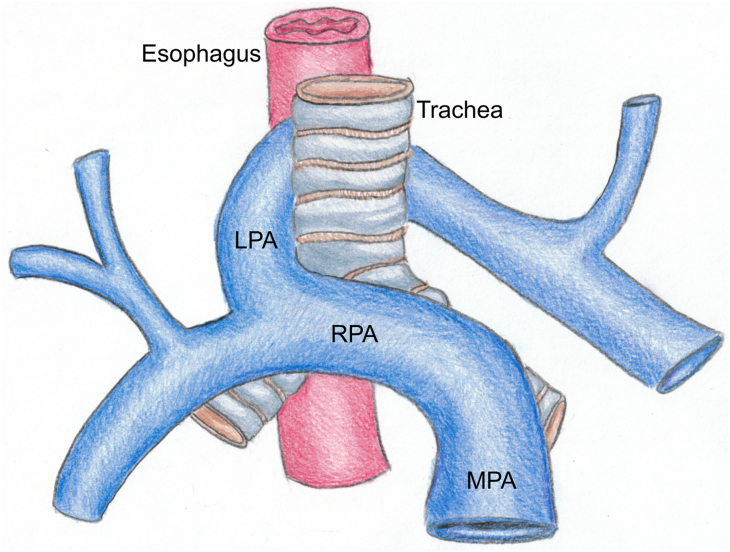
Anatomy of a left pulmonary artery (LPA) sling. In LPA sling, the LPA arises anomalously from the right pulmonary artery (RPA) instead of the main pulmonary artery (MPA). It courses between the trachea and esophagus, potentially compressing one or both structures. Used with permission of Susan Rayner.

A 63-year-old woman was referred to the University of Washington for progressive airway and esophageal symptoms in the setting of a known congenital LPA sling. She had been diagnosed at birth with LPA sling and spent months in intensive care as a neonate. She suffered from lifelong intermittent cough, wheezing, and stridor, leading to admissions for “asthma” in childhood, although she maintained a good functional status and was able to participate in sports during high school, albeit with some difficulty.

Her symptoms worsened in adulthood and particularly progressed during the 10 years before referral. On evaluation at our institution, she reported dyspnea, wheezing, orthopnea, dysphagia, and exertional syncope. Pulmonary examination was notable for stridor with faint biphasic wheeze. Computed tomography (CT) demonstrated LPA sling with significant tracheal and esophageal compression (Figure 2A) as well as mild aneurysmal dilation of the main and branch pulmonary arteries that had progressed compared with imaging obtained 9 years prior. Cardiac catheterization identified normal pulmonary artery pressure and pulmonary vascular resistance. Pulmonary angiography confirmed LPA sling.

**Figure 2:**
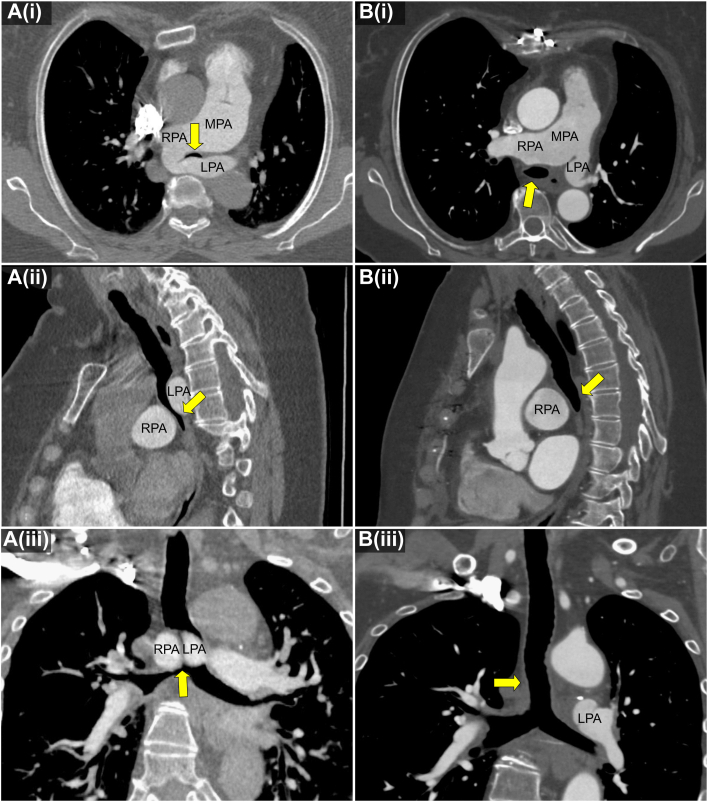
Computed tomography before and after left pulmonary artery sling repair. (A) Preoperative computed tomography shows significant tracheal compression (arrow) between the right and left pulmonary arteries, as visualized in (i) axial, (ii) sagittal, and (iii) coronal planes. (B) Postoperative imaging shows marked improvement in tracheal patency at the site of prior compression (arrow). (LPA, left pulmonary artery; MPA, main pulmonary artery; RPA, right pulmonary artery.)

After multidisciplinary discussion, surgical LPA translocation was offered. Preoperative bronchoscopy revealed severe tracheal compression at the level of the LPA sling, visually estimated at 80% narrowing, but without associated tracheobronchial abnormality that might necessitate combined tracheal reconstruction. She underwent successful operative repair of the LPA sling under cardiopulmonary bypass through median sternotomy, without circulatory arrest. The LPA was dissected where it passed behind the trachea and in front of the esophagus, and the ligamentum arteriosum was ligated and divided to improve mobilization of the pulmonary artery. The LPA was shortened to avoid kinking of the vessel and was reimplanted onto the main pulmonary artery. Flexible bronchoscopy demonstrated marked improvement in tracheal opening. Her postoperative course was unremarkable. In the postoperative period, care was taken to maintain normotension, with a target systolic blood pressure of <130 mm Hg and mean arterial pressure of >65 mm Hg. Whereas anticoagulation was not required, the patient was prescribed aspirin 81 mg on postoperative day 1, which was continued for 6 months.

The patient was seen in pulmonary clinic 6 months after surgery. She reported resolution of presyncope and dysphagia and improvement in her exertional dyspnea. Examination was notable for absence of stridor and wheeze. She continues to report productive cough, unchanged after surgery. Follow-up chest CT showed resolution of tracheal compression (Figure 2B). Pulmonary function testing demonstrated normalization of spirometry after surgery. Forced expiratory volume in 1 second increased from 1.73 L (65% predicted) preoperatively to 2.58 L (111% predicted) postoperatively, forced vital capacity increased from 2.91 L (84% predicted) to 3.05 L (104% predicted), and the forced expiratory volume in 1 second/forced vital capacity ratio improved from 0.59 to 0.79. Obstructive morphology on the flow-volume loop also resolved (Figure 3).

**Figure 3:**
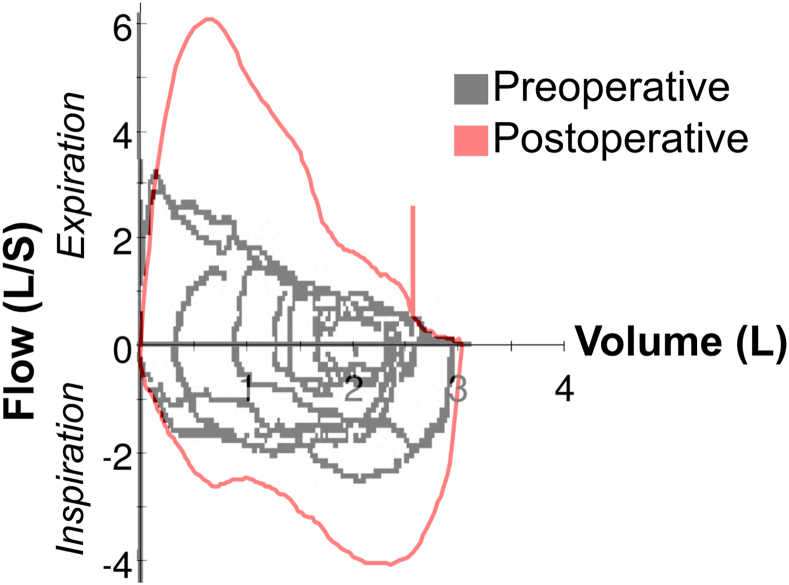
Pulmonary function testing. Preoperative flow-volume loops (gray) demonstrate reduced inspiratory and expiratory flow, with flattening of the inspiratory limb consistent with central airway compression. Postoperative testing (red) shows improved inspiratory and expiratory flow, reflecting relief of airway obstruction. L, litres; L/S, litres/second.

## Comment

This is a unique report describing surgical repair of LPA sling in an adult for symptoms of progressive airway obstruction. A prior report discussed successful resolution of dysphagia with adult LPA sling repair, but the effects on pulmonary function were not reported.^4^

The decision to pursue surgical repair for relief of airway obstruction in the setting of LPA sling is complex. Patients with LPA sling often have airway symptoms due to associated tracheobronchial abnormalities, including complete tracheal rings and/or tracheal stenosis, rather than purely from obstruction by a vascular sling—situations that may not be improved by vascular repair alone, without concomitant tracheoplasty.^6^^,^^7^ Especially pertinent to pediatric cases, LPA slings can be classified by the position of the LPA relative to the carina and the presence or absence of airway abnormalities.^8^ Type I slings pass above the carina and are subdivided into IA (normal tracheobronchial tree) and IB (right tracheal bronchus present). Type II slings pass below the carina and are often associated with complex airway abnormalities. In this case, no associated tracheobronchial or other congenital abnormalities were present, consistent with type IA LPA sling, and symptoms were attributed to vascular impingement on the trachea. We hypothesized that the progressive dilation of her pulmonary arteries during the preceding decade, seen on CT imaging, may have contributed to her worsening tracheal compressive symptoms. It was initially unclear to what extent tracheal function would recover after lifelong compression or whether she might be left with tracheomalacia despite vascular correction. However, in the face of severe and progressive compressive symptoms to the point of syncope, we thought a trial of surgery was indicated.

Surgery was approached through sternotomy in this adult patient for optimal exposure of the pulmonary arteries and surrounding structures; thoracotomy may be an option for smaller adults, especially those requiring concomitant tracheal repair. Whereas pulmonary compromise was the main indication for her operation, she did have dysphagia and CT evidence of esophageal compression, both of which improved postoperatively. In future cases with predominant foregut symptoms, additional preoperative esophageal evaluation may be useful, as previously reported.^4^

Overall, both the patient and her treatment team were pleased with her symptomatic and functional outcome, although we suspect her ongoing cough may be related to residual tracheobronchial dysfunction. In summary, this case demonstrates that relief of obstructive pulmonary physiology and excellent clinical outcomes are possible in selected adults with LPA sling. We also highlight the importance of careful anatomic and physiologic assessment to guide intervention for these patients.
